# Molecular Mechanisms of Obesity-Induced Development of Insulin Resistance and Promotion of Amyloid-β Accumulation: Dietary Therapy Using Weak Organic Acids via Improvement of Lowered Interstitial Fluid pH

**DOI:** 10.3390/biom13050779

**Published:** 2023-04-30

**Authors:** Yoshinori Marunaka

**Affiliations:** 1 Medical Research Institute, Kyoto Industrial Health Association, Kyoto 604-8472, Japan; marunaka@koto.kpu-m.ac.jp; 2 Research Organization of Science and Technology, Ritsumeikan University, Kusatsu 525-8577, Japan; 3 Graduate School of Medical Science, Kyoto Prefectural University of Medicine, Kyoto 602-8566, Japan

**Keywords:** insulin resistance, interstitial fluid pH, type 2 diabetes mellitus, amyloid-β, Alzheimer’s disease, weak organic acids, SMCT1, pH capacity, acidosis, ketone bodies

## Abstract

Insulin resistance is one of the etiologies of type 2 diabetes mellitus (T2DM) and has been suggested to contribute to the development of Alzheimer’s disease by promoting amyloid-β accumulation. Various causes of insulin resistance have been suggested; however, mechanisms of insulin resistance development remain to be elucidated in many respects. Elucidating the mechanisms underlying the development of insulin resistance is one of the key factors in developing methods to prevent the onset of T2DM and Alzheimer’s disease. It has been suggested that the body pH environment plays an important role in the control of cellular functions by regulating the action of hormones including insulin and the activity of enzymes and neurons, thereby maintaining homeostatic conditions of the body. This review introduces: (1) Mitochondrial dysfunction through oxidative stress caused by obesity-induced inflammation. (2) Decreased pH of interstitial fluid due to mitochondrial dysfunction. (3) Development of insulin resistance due to diminution of insulin affinity to its receptor caused by the lowered interstitial fluid pH. (4) Accelerated accumulation of amyloid-β due to elevated activities of β- and γ-secretases caused by the lowered interstitial fluid pH. (5) Diet therapies for improving insulin resistance with weak organic acids that act as bases in the body to raise the pH of lowered interstitial fluid and food factors that promote absorption of weak organic acids in the gut.

## 1. Introduction

Insulin resistance is characterized by impairment of insulin action in insulin-targeting tissues and organs such as skeletal muscles, adipocytes and the liver, and causes hyperglycemia observed in type 2 diabetes mellitus (T2DM) [1,2]. Continued food intake in excess of requirements causes obesity via hypertrophy and hyperplasia of adipocytes in white adipose tissue (WAT) [3], which is suggested to cause insulin resistance [3]. The hypertrophy of adipocytes produces proinflammatory responses leading to relatively mild chronic inflammation in adipose tissues via physical expansion of adipocyte size [4,5]. This inflammation causes oxidative stress associated with oxidation of DNA, lipids and proteins [6,7,8], which has been suggested to induce insulin resistance via mitochondrial dysfunction [2]. However, it is still unclear how mitochondrial dysfunction causes insulin resistance.

On the one hand, it has been reported that insulin resistance causes the overproduction of free fatty acids, releasing pro-inflammatory cytokines such as tumor necrosis factor-α (TNF-α) and nuclear factor-κB (NF-κB) into the blood circulation [9]. These cytokines further induce activation of serine kinases, IkB kinase (IKK) and c-Jun N-terminal kinase (JNK) [10], thereby inhibiting insulin signaling via the phosphorylation of insulin receptor substrate-1 (IRS-1), blocking activation of insulin receptor substrate (IRS) proteins by binding to the phosphorylated insulin receptor and promoting IRS degradation by ubiquitination [9]. However, it is still unclear if insulin resistance first occurs due to any cause, then induces inflammation via the overproduction of free fatty acids, leading to further dysfunction of insulin signals associated with mitochondrial disorders [9].

Mitochondrial dysfunction [11] results in impaired aerobic glucose metabolism, reducing efficiency of energy (ATP) production [11]. In a state of mitochondrial dysfunction, it becomes necessary to consume more glucose or promote non-glucose metabolism, such as lipids, to maintain ATP production in the amount necessary to sustain vital activities. This situation increases acid production, lowering the pH of the interstitial fluid, which has a pH capacity much smaller than that of blood and the intracellular fluid [12]. As the interstitial fluid pH decreases, the insulin affinity to its receptor is diminished, causing insulin resistance [12].

T2DM patients have an increased risk of developing mental disorders including dementia via neurological dysfunction caused by microvascular complications of diabetes [13]. In addition, patients with T2DM have a higher risk of developing Alzheimer’s disease, another type of dementia [14], due to the development of insulin resistance [14,15,16]. Accumulation of amyloid-β is considered to be one of the main causes of Alzheimer’s disease [17,18], although accumulation of amyloid-β has not yet been confirmed to directly cause Alzheimer’s disease [17,18]. In a state of insulin resistance, the insulin concentration increases. Insulin is a substrate of neprilysin, an amyloid-β-degrading enzyme. Therefore, neprilysin exclusively degrades insulin in the insulin-resistant state, thus reducing the degree of amyloid-β degradation [19]. This leads to accelerated accumulation of amyloid-β in the state of insulin resistance, suggesting that insulin resistance would be a cause of Alzheimer’s disease [15]. Furthermore, acidic conditions elevate the activity of β- and γ-secretases [20,21,22,23,24,25], enzymes that produce amyloid-β from amyloid precursor protein [19,26], resulting in increased amyloid-β production. Thus, the lowered pH of the interstitial fluid observed in insulin resistance accelerates the accumulation of amyloid-β.

Weak organic acids containing carboxyl groups behave as bases inside the body after absorption via Na^+^-coupled carboxylate transporters (SCT) across the gut [27]. This suggests that intake of weak organic acids improves the lowered interstitial fluid pH via elevation of pH buffer capacity [12,27], leading to amelioration of insulin resistance [12]. In addition, it should be considered that increasing the efficiency of absorption of weak organic acids in the gut is a beneficial way to elevate the lowered interstitial fluid pH.

This narrative review provides evidence for molecular mechanisms regarding development of insulin resistance and comprehensively discusses the following points: (1) Mitochondrial dysfunction due to oxidative stress resulting from obesity-induced inflammation. (2) Lowered interstitial fluid pH due to mitochondrial dysfunction. (3) Development of insulin resistance caused by diminution of insulin affinity to its receptor caused by the lowered interstitial fluid pH. (4) Accelerated accumulation of amyloid-β due to elevated activities of β- and γ-secretases via the lowered interstitial fluid pH. (5) Diet therapies for improving insulin resistance with weak organic acids containing carboxyl groups acting as bases in the body to raise lowered interstitial fluid pH and food factors promoting absorption of weak organic acids in the gut.

To obtain the information on subjects of this review article, I have searched articles providing molecular mechanisms of insulin resistance onset by using the following terms: insulin resistance, interstitial fluid pH, type 2 diabetes mellitus, amyloid-β, Alzheimer’s disease, β- and γ-secretases, weak organic acids, SMCT1 (Na^+^-coupled monocarboxylate transporter 1), pH capacity, acidosis, ketone bodies, lactic acid and diet therapies in PubMed and Google Scholar.

## 2. Types of Adipose Tissues and Their Roles in Obesity

Obesity is recognized as a trigger for development of insulin resistance. It is well known that dietary intake in excess of energy expenditure required to maintain vital activities leads to obesity, which is stored in adipose tissues as fat [28,29,30]. Mammals have three types of adipose tissues: (1) white adipose tissue (WAT), (2) brown adipose tissue (BAT) and, (3) beige adipose tissue (BeAT) [28,30]. WAT and BAT are classically recognized, while BeAT has been found recently. WAT, the majority of adipose tissues, contains white adipocytes with unilocular lipid droplets and scarce mitochondria, and plays a role as the primary site of energy (lipid) storage [29]. In contrast, BAT contains brown adipocytes displaying multilocular lipid droplets with a high number of mitochondria, and has thermogenic ability via elevation of uncoupling protein 1 (UCP1) amounts in the inner membrane mitochondria [29]. In BAT, utilization of a high number of mitochondria via elevation of UCP1 leads to uncoupled oxidative phosphorylation from ATP, dissipating chemical energy as heat [29]. Therefore, BAT influences the entire body metabolism, and has the ability to modify the susceptibility to increasing body weight. In humans, BAT was thought to be an energy-producing tissue only in newborns and to regress with age [29]. However, in human adults, BAT has also been identified around the aorta and within the neck supraclavicular region [29]. Furthermore, BeAT, a recently identified type of adipose tissue, is confirmed to have structural and functional features of both WAT and BAT [31,32,33]. BeAT is thought to be generated via conversion of WAT into BeAT and de novo differentiation from a progenitor resident cell [28,29]. Thus, WAT plays a role as the primary site of energy storage, while BAT and BeAT participate in energy expenditure and thermogenesis, although BeAT may also contribute to energy storage. Thus, WAT is more closely linked to the metabolic complications of obesity, such as diabetes.

## 3. Obesity-Induced Release of Pro-Inflammatory Cytokines and Mitochondrial Damage

**Figure 1:**
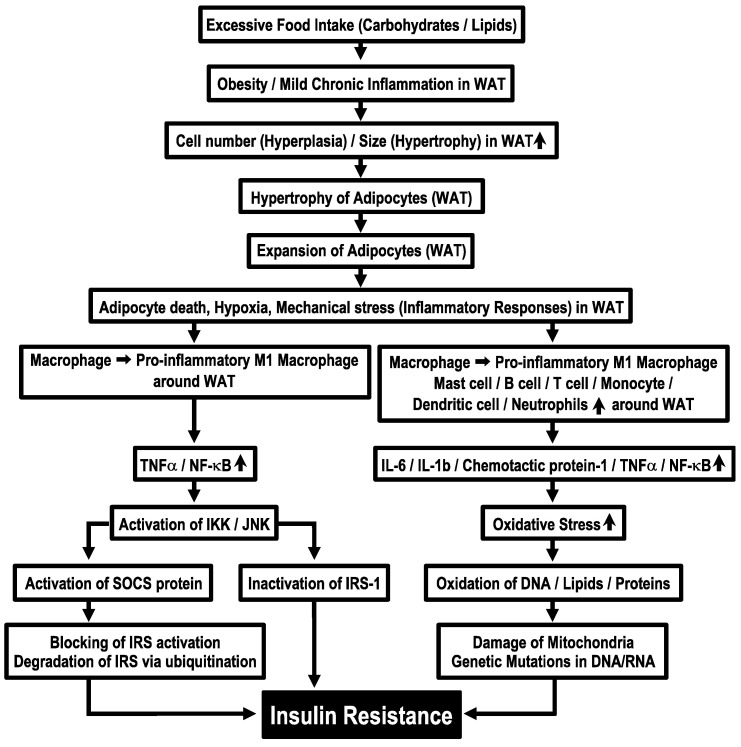
Molecular mechanisms of obesity-induced insulin resistance. WAT, white adipose tissue.

Obesity due to continuous dietary intake in excess of energy expenditure is associated with hyperplasia and hypertrophy of adipocytes in WAT [34] and causes excessive release of free fatty acids [35,36]. In the circumstance of adipose tissues, macrophages are polarized into pro-inflammatory M1 macrophages [3], which release pro-inflammatory cytokines, TNF-α and NF-κB [9]. These cytokines activate serine kinases, IKK and JNK [10], which inactivate insulin signals by phosphorylating IRS-1 at serine-302 (pS302) and serine-307 (pS307), instead of its normal tyrosine residue phosphorylation site [9]. IKK and JNK activate further suppressors of cytokine signaling (SOCS) protein, an inflammatory-related negative regulator of IRS proteins, blocking activation of IRS proteins by binding with the phosphorylated insulin receptor and promoting degradation of IRS via ubiquitination [9]. These phenomena induce insulin resistance (Figure 1). Furthermore, immune cells, such as dendritic cells, mast cells, B cells, T cells and neutrophils, appear around WAT under the condition of sustained energy supply in excess of requirements [34]. These phenomena increase levels of proinflammatory cytokines such as interleukin-6 (IL-6), interleukin-1β (IL-1β), chemotactic protein-1 and TNF-α [5] and also produce more oxidative stress [5]. This process further leads to oxidation of DNA, lipids and proteins, which causes mitochondria damage and genetic mutations in DNA/RNA [6] (Figure 1). Derivatives of oxidative reactions are reported to increase in insulin resistance [37]. However, the molecular mechanisms of how mitochondrial damage and DNA/RNA genetic mutations cause the development of insulin resistance remain to be elucidated.

## 4. Onset of Insulin Resistance due to Lowered Interstitial Fluid pH Caused by Mitochondrial Damage

**Figure 2:**
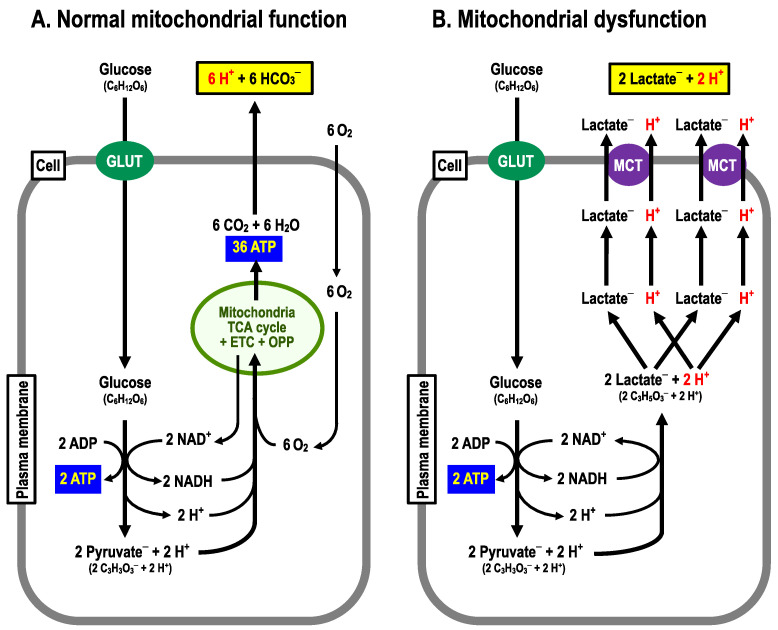
Metabolizing pathways. (**A**) Normal mitochondrial function. From 1 molecule of glucose, 38 molecules of ATP, 6 moles of CO_2_ are produced by consuming 6 molecules of O_2_. (**B**) Mitochondrial dysfunction. From one molecule of glucose, two molecules of ATP and two equivalents of H^+^ are produced.

As is well known, glucose metabolism is performed via the anaerobic process in the cytoplasm by the glycolytic pathway followed by the aerobic process in mitochondria through the Krebs (TCA) cycle associated with the following two processes: (i) the electron transport chain (ETC) and (ii) the oxidative phosphorylation pathway (OPP; a H^+^ channel (F0) and ATPase (F1)) [38]. Cells with normal function of mitochondria produce 38 molecules of ATP, 6 molecules of CO_2_ and 12 molecules of H_2_O from 1 molecule of glucose, consuming 6 molecules of O_2_ and 6 molecules of H_2_O with no net production of H^+^ under aerobic conditions (Figure 2A) [39,40]. On the one hand, only two molecules of ATP are produced from one molecule of glucose with production of two molecules of lactate^–^ and two equivalents of H^+^ via the glycolysis process in an anaerobic or mitochondrial dysfunctional state (Figure 2B) [39,40]. Thus, to obtain the ATP needed to sustain vital activity, this state requires the metabolism of far more molecules of glucose than normal mitochondrial function, where O_2_ is available, producing much more lactate^–^ and H^+^ [27], which are extruded to the extracellular space (interstitial fluid space) via monocarboxylate transporter (MCT) (Figure 2B). Therefore, it is necessary to consider how the pH condition in a state of mitochondrial dysfunction differs from that in a state of normal mitochondrial function.

**Figure 3:**
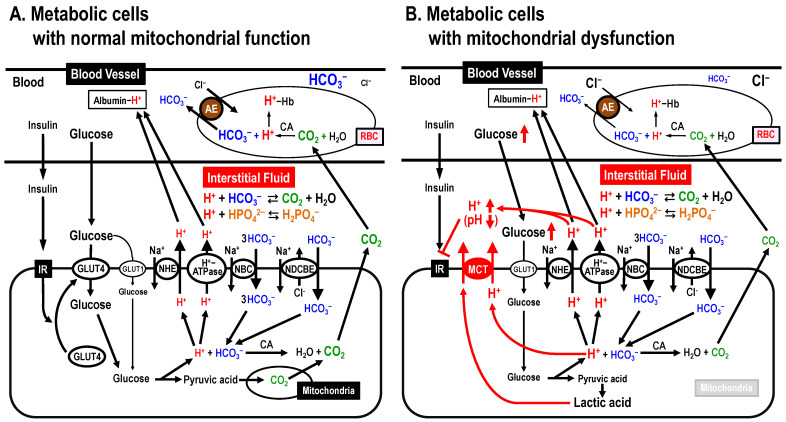
Mitochondrial dysfunction-induced insulin resistance via lowering interstitial fluid pH. (**A**) Metabolic cells with normal mitochondrial function. (**B**) Metabolic cells with mitochondrial dysfunction. AE, anion exchanger; CA, carbonic anhydrase; MCT, monocarboxylate transporter; NBC, Na^+^-HCO_3_^–^ cotransporter; NDCBE, Na^+^-driven Cl^–^/HCO_3_^–^ exchanger; NHE, Na^+^/H^+^ exchanger. Modified from Figure 3 in *Int. J. Mol. Sci.* **2018**, *19*, 3244 ([12]).

One pH homeostatic system commonly functioning in the blood, cytosolic space and interstitial fluid is the pH buffering system via bicarbonate-carbonate (HCO_3_^–^-CO_2_)- and phosphoric acid (H_3_PO_4_) [41] (Figure 3A). Blood contains H^+^-binding proteins, such as hemoglobin and albumin, strong pH buffers, keeping blood pH within the narrow normal range (7.35~7.45), and proteins in the intracellular space there also exhibit pH buffering capacity (Figure 3A). In addition, H^+^ transporters extrude H^+^ to the extracellular space (the interstitial fluid space) across the plasma membrane. On the one hand, the interstitial fluid has little pH buffer capacity compared with blood and/or the intracellular space, thus the interstitial fluid pH is likely to be variable compared with the pH of blood and/or the intracellular space. In fact, the interstitial fluid pH in T2DM patients with mitochondrial dysfunction is lower than that in healthy individuals (Figure 3B) [11,14,42,43,44,45,46,47,48]. 

The pH of fluids around an enzyme or substrate can alter the binding affinity of the enzyme and substrate, which in turn changes the activity of the enzyme [49]. This is because H^+^-bindings dramatically change the protein tertiary structure, and H^+^ also acts on key residues in the catalytic site of the enzyme [49]. pH alteration also changes the binding affinity of insulin and its receptor. Indeed, lowered pH in the interstitial fluid has been reported to diminish the binding affinity of insulin and its receptor, leading to insulin resistance (Figure 3B) [50,51].

Thus, maintenance of the interstitial fluid pH within normal ranges is essential for prevention of pathogenesis of insulin resistance. Strategies for maintenance of the interstitial fluid pH are described later in this article.

## 5. Insulin Resistance Caused by Lowered pH of the Interstitial Fluid

### 5.1. Molecular Mechanisms of Co-Occurrence of Insulin Resistance and Chronic Obstructive Pulmonary Disease (COPD) via Lowered pH of the Interstitial Fluid

The CO_2_ produced in mitochondria of metabolic cells moves into red blood cells (RBC), where CO_2_ is transformed to H^+^ and HCO_3_^–^ via a CA-facilitated process. H^+^ binds to Hb, while HCO_3_^−^ is exchanged for extracellular Cl^–^ by an anion exchanger (AE; Cl^–^/HCO_3_^–^ exchanger) and excreted out of RBC (Figure 3A). Then, blood moves to the lung via the heart. In the lung, the partial pressure of CO_2_ is much lower than in environments around peripheral metabolic cells. Therefore, H^+^ and HCO_3_^–^ are transformed to CO_2_ and H_2_O via a CA-facilitated process in the lung. Then, CO_2_ moves to the atmosphere via alveolar cells participating in gas exchange (CO_2_/O_2_ exchange). COPD patients with disturbances in CO_2_/O_2_ exchange in alveolar cells due to alveolar cell damages show high CO_2_ partial pressure leading to acidic conditions, especially in the interstitial fluid [52]. Multiple cell types including neutrophils in COPD patients produce large amounts of tumor necrosis factor-α (TNF-α) [53], which is one of the factors causing insulin resistance [53]. Thus, COPD patients are at high risk of developing insulin resistance via a TNF-α-mediated pathway [52,54]. However, it should also be considered that the interstitial fluid acidity due to elevated CO_2_ level in COPD patients [55,56,57,58] would cause insulin resistance [12].

### 5.2. Molecular Mechanisms of Insulin Resistance Development due to High Salt Intake via Lowered pH of the Interstitial Fluid

High salt (Na^+^) intake has been reported to be one of the pathogeneses of insulin resistance [59,60,61,62], although little information is available on the molecular mechanism by which high salt intake causes insulin resistance. High salt (Na^+^) intake leads to an excessive increase in Na^+^ content in the intravascular and interstitial spaces. High Na^+^ contents in the interstitial fluid increase the chemical potential of Na^+^ in the interstitial fluid, resulting in an excess supply of Na^+^ into the intracellular space. Cells overloaded with Na^+^ attempt to excrete Na^+^ out of the cells by Na^+^,K^+^-ATPase (Na^+^,K^+^-pump), consuming large amounts of ATP. This process lowers the interstitial fluid pH by producing large amounts of H^+^ [12], leading to insulin resistance [63,64,65,66], although more experimental evidence is required to confirm this process.

### 5.3. Molecular Mechanisms of Co-Occurrence of Insulin Resistance and Ketone Body/Lactic Acid Production via Lowered pH of the Interstitial Fluid

Ketone bodies, other major H^+^ sources, are produced from fatty acids under glucose-unavailable pathophysiological conditions in the liver [67] due to insulin resistance and failure of insulin secretion from pancreatic β cells [68,69]. It is notable that even under physiological conditions, ketone bodies are produced by prolonged exercise or extremely low-carbohydrate diet [70,71,72,73]. β-hydroxybutyric acid (β-hydroxybutyrate^−^ + H^+^) is one of the major ketone bodies [70,71,72,73]. Under glucose-unavailable conditions, β-hydroxybutyric acid serves as an energy source in extrahepatic tissues/organs with normal mitochondrial function [70,71,72,73]. β-hydroxybutyric acid exists as an ionized form, β-hydroxybutyrate^−^ + H^+^, around normal intracellular pH value, approximately 7.2~7.6, much larger than the pKa (~4.8) of β-hydroxybutyric acid [74].

High production of β-hydroxybutyric acid in hepatocytes leads to lowered intracellular fluid pH even in healthy persons on extremely low-carbohydrate diets or performing prolonged exercise, as well as T2DM patients with mitochondrial dysfunction [71]. This means that extremely low-carbohydrate diets carry a high risk of causing pathologies such as insulin resistance and amyloid-β accumulation (see the description in the following section in detail), therefore we should pay attention to risks generated from extremely low-carbohydrate diets.

Metformin, a biguanide compound, has been developed as a drug inhibiting gluconeogenesis in the liver for reduction of blood glucose levels [75,76,77,78,79,80,81,82,83], since the elevated blood glucose level is one of the most typical symptoms observed in T2DM patients, causing various types of damages including cerebral and myocardial infarctions, and susceptibility to infection [79,84]. Metformin suppresses hepatic gluconeogenesis via inhibition of complex I of the electron transport chain in mitochondria [75,85], resulting in elevation of lactic acid production (Figure 2B) [75,85]. Thus, metformin may induce lactic acidosis [75,85,86,87,88,89], diminishing the insulin action. Imeglimin is also developed for reduction of blood glucose levels: it has a similar structure to metformin, but contains a triazine ring unlike metformin [75,90]. Imeglimin does not cause lactic acidosis unlike metformin, and is characterized by its ability to rebalance respiratory chain activity via partial inhibition of Complex I and correction of deficient Complex III activity, thereby reducing reactive oxygen species (ROS) production and ameliorating insulin resistance [75,90]. In addition, imeglimine is reported to show better glycemic control when used in combination with metformin in T2DM patients [75].

Inhibitors of sodium-dependent glucose transporter 2 (SGLT2), which is involved in sodium-dependent glucose reabsorption in the renal proximal tubules, have also been developed [75,79,82,84]. Although SGLT2-inhibitors are very useful drugs reducing blood glucose levels [75,79], unfortunately some T2DM patients taking SGLT2-inihibitors [79,83,91,92,93,94] show ketoacidosis via generation of ketone bodies such as β-hydroxybutyric acid due to extremely low glucose availability caused by SGLT2-inhibitors [75,79,82,84,95,96], resulting in severe insulin resistance.

## 6. Accumulation of Amyloid-β Caused by Lowered pH of the Interstitial Fluid in Insulin Resistance

Accumulation of amyloid-β is well known to occur in patients suffering with Alzheimer’s disease [97,98,99,100,101]. Although accumulation of amyloid-β is not yet confirmed to directly cause Alzheimer’s disease [17,18], it is obvious that accumulation of amyloid-β is one of the main causes of Alzheimer’s disease [17,18]. Accumulation of amyloid-β has been also reported to induce hyperphosphorylation of tau protein, causing neural inflammation, neural loss and synaptic impairment associated with cognitive decline and behavioral abnormalities [102].

T2DM patients are at high risk of developing Alzheimer’s disease [103,104,105]. A possible explanation for the higher risk of developing Alzheimer’s disease in T2DM is that: (1) Neprilysin, an enzyme degrading amyloid-β, has another target protein for degradation, insulin; (2) T2DM patients exhibit hyperinsulinemia; (3) in T2DM patients with hyperinsulinemia, neplilysin contributes to degradation of insulin in addition to amyloid-β, resulting in less amyloid-β degradation by neplilysin than in healthy individuals; (4) this process increases the accumulation of amyloid-β [106,107,108]. This explanation is currently well accepted by many medical researchers and physicians. However, activities of β- and γ-secretases, which are involved in the formation of amyloid-β from amyloid precursor protein [19,26], should also be considered. Activities of β- and γ-secretases are known to be elevated by acidic conditions [20,21,22,23,24,25]. Thus, the acidic condition that occurs in T2DM patients increases the production of amyloid-β by elevating activities of β- and γ-secretases [17,18,26,102]. Further, the effects of acidic conditions on the activity of neprilysin, an enzyme degrading amyloid-β, should also be considered [81,100,106,107,109]. It has been reported that neprilysin activity is reduced in acidic environments [110], suggesting that amyloid-β accumulation is enhanced. Thus, the acidic environment observed in T2DM promotes the β- and γ-secretase-facilitated production of amyloid-β and diminishes the neprilysin-facilitated degradation of amyloid-β, leading to elevated amyloid-β accumulation.

We will also consider the relationship between amyloid-β accumulation and high salt intake induced hypertension. Some reports have shown a relationship between salt-sensitive hypertension and Alzheimer’s disease [111,112]. However, little information is available on how these diseases are correlated. One explanation is that the lower interstitial fluid pH observed in salt-sensitive hypertension promotes the accumulation of amyloid-β by increasing the activity of β- and γ-secretases and decreasing the activity of neprilysin [12].

## 7. Ameliorating Action of Food Compounds on Insulin Resistance and Accumulation of Amyloid-β

Amelioration (elevation) of lowered interstitial pH would be one of the most intrinsically effective therapeutics for insulin resistance in T2DM. A possible method ameliorating the lowered interstitial fluid pH would be intake of weak organic acids [12]. Weak organic acids, such as citric and acetic acids, are tasted as sour substances on the tongue, since they contain H^+^ [27], while weak organic acids behave as bases in the body after absorption via Na^+^-coupled carboxylate transporters (SCT) [113,114], which transport only the carboxyl groups but not H^+^ [113,114]. Therefore, absorbed dietary weak organic acids behave as bases but not acids, elevating the pH buffering capability to ameliorate (elevate) the lowered interstitial fluid pH in T2DM patients. Thus, intake of weak organic acids is a useful way to ameliorate insulin resistance [12].

Furthermore, efficient absorption of dietary weak organic acids should be considered. Brazilian propolis, ninjin’yoeito and mumefural have been reported to ameliorate insulin resistance in T2DM by elevating the lowered interstitial fluid [63,115,116]. Mumefural increases the expression of SMCT1, a carboxylate transporter, in the colon [116], and ninjin’yoeito also tends to have a similar effect [115]. Thus, by increasing the expression of Na^+^-coupled carboxylate transporters, these compounds elevate the absorbing amount of carboxyl groups, which behave as bases in the body. Therefore, Brazilian propolis, ninjin’yoeito and mumefural ameliorate insulin resistance via an increase in the interstitial fluid pH by elevating the efficiency of carboxyl group absorption. Amelioration of insulin resistance essentially participates in prevention from frailty by stimulating glucose uptake into skeletal muscles [117,118,119,120]. Thus, dietary therapy combining weak organic acids with compounds that enhance the functional expression of weak organic acid transporters is one useful, efficient T2DM treatment strategy that promotes glucose uptake into skeletal muscles, especially for elderly T2DM patients with high risk of frailty. This combining therapy would also be useful in preventing amyloid-β accumulation via decreased activity of β- and γ-secretases and elevated activity of neprilysin.

In addition to ameliorating interstitial fluid pH, ninjin’yoeito is known to improve glucose metabolism in insulin-sensitive tissues via the brainstem Y4 circuit [121]. Brazilian propolis has also been reported to prevent diabetes and obesity by promoting glucagon-like peptide-1 (GLP1) secretion [122]. Further, mumefural has anti-diabetic properties by inhibiting lipogenesis and inducing BAT/BeAT [123]. Thus, Brazilian propolis, ninjin’yoeito and mumefural would ameliorate insulin resistance through coordinated action of the GLP1/brainstem-Y4-circuit/anti-lipogenesis pathways and interstitial fluid pH improvement.

## 8. Conclusions

**Figure 4:**
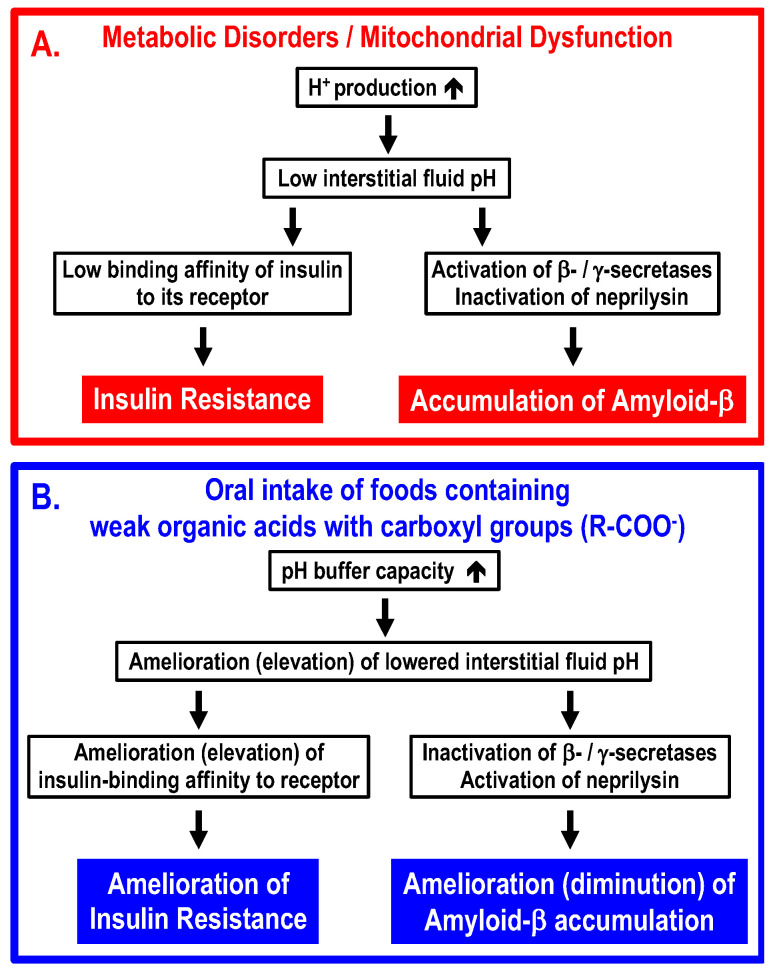
Molecular mechanisms by which metabolic disorders and/or mitochondrial dysfunction lower interstitial fluid pH, leading to insulin resistance and accumulation of amyloid-β (Panel A), and ameliorating processes by oral intake of foods containing weak organic acids with carboxyl groups (Panel B). (**A**) Under conditions with metabolic disorders and/or mitochondrial dysfunction, (1) large amounts of protons (H^+^) are produced in metabolic cells, (2) the large amounts of protons (H^+^) produced in metabolic cells decrease the interstitial fluid pH due to limited pH-buffering capacity, (3) the lowered interstitial fluid pH diminishes the insulin-binding affinity to its receptor, increases activities of β-/γ-secretases and decreases activities of neprilysin, and (4) the diminution of insulin-binding affinity to its receptor develops insulin resistance, and increased activities of β-/γ-secretases and decreased activity of neprilysin promote accumulation of amyloid-β. (**B**) Oral intake of foods containing weak organic acids increases the pH capacity of interstitial fluids, ameliorating (elevating) the lowered pH of the interstitial fluid. The amelioration (elevation) of the lowered interstitial fluid pH increases insulin-binding affinity to its receptor, decreases activities of β-/γ-secretases and increases activities of neprilysin, leading to amelioration of insulin resistance and diminution of amyloid-β accumulation.

The conclusion of the present article is summarized in Figure 4. Interstitial fluid pH is very variable, since the pH-buffering capacity of interstitial fluids is very limited. Because of the limited pH buffering capacity, even if the cytoplasmic space and “arterial” blood are maintained in the normal pH range, the interstitial fluid can easily become acidic if an excess of acidic metabolites are produced in the metabolizing cells. One of the most serious problems caused by lowered interstitial fluid pH is the development of insulin resistance via decreased binding affinity of insulin to its receptor. Another problem caused by lowered interstitial fluid pH would be an increase in accumulation of amyloid-β via elevation of amyloid-β-producing β-/γ-secretase activities and diminution of amyloid-β-degrading neprilysin activity. Intake of foods containing weak organic acids ameliorates (elevates) the lowered interstitial fluid pH in T2DM patients, rescuing T2DM patients from insulin resistance via elevation of insulin binding affinity to its receptor, and ameliorates (diminishes) the accumulation of amyloid-β by regulating activities of β-/γ-secretases and neprilysin. Further, it is essentially important to discover compounds, such as mumefural, that upregulate the expression of Na^+^-coupled carboxylate transporters involved in the uptake of carboxyl groups contained in weak organic acids via epithelia of the intestine and/or the colon into the body.

## Data Availability

Not applicable.
